# Yield of esophagogastroduodenoscopy and colonoscopy in cancer of unknown primary

**DOI:** 10.12669/pjms.292.3212

**Published:** 2013-04

**Authors:** Muhammad Tayyab Usmani, Abdullah Bin Khalid, Syed Hasnain Ali Shah, Tauseef Ahmad, Saeed S. Hamid, Syed M Wasim Jafri

**Affiliations:** 1 Muhammad Tayyab Usmani, Section of Gastroenterology, Department of Medicine, Aga Khan University Hospital, Stadium Road, PO Box # 74800, Karachi, Pakistan.; 2 Abdullah Bin Khalid, Dow University of Health Sciences, Karachi, Pakistan.; 3 Syed Hasnain Ali Shah, Section of Gastroenterology, Department of Medicine, Aga Khan University Hospital, Stadium Road, PO Box # 74800, Karachi, Pakistan.; 4 Tauseef Ahmad, Section of Gastroenterology, Department of Medicine, Aga Khan University Hospital, Stadium Road, PO Box # 74800, Karachi, Pakistan.; 5 Saeed S. Hamid, Section of Gastroenterology, Department of Medicine, Aga Khan University Hospital, Stadium Road, PO Box # 74800, Karachi, Pakistan.; 6 Syed M. Wasim Jafri, Section of Gastroenterology, Department of Medicine, Aga Khan University Hospital, Stadium Road, PO Box # 74800, Karachi, Pakistan.

**Keywords:** Esophagogasrtoduodenoscopy (EGD), Colonoscopy, Cancer of unknown primary (CUP)

## Abstract

***Objectives: ***Carcinoma of unknown primary origin (CUP) is heterogeneous group of cancers. Role of gastrointestinal (GI) endoscopy in this entity is under investigated. Aim of this study was to evaluate yield of Colonoscopy and Esophagogastroduodenoscopy (EGD) in localizing primary tumor in patients with CUP.

***Methodology: ***Patients with histopathologically proven CUP who underwent colonoscopy / EGD to find the primary tumor from December 2009 to December 2011 were included in the study. Abdominal symptoms and cytokeratin (CK) 7 and 20 markers were correlated with presence of primary in GI tract.

***Results: ***After giving informed consent 86 patients were included in final analysis. All patients underwent colonoscopy while 60(70%) got EGD along with colonoscopy. Mean age was 55.10 +/-11.94 years with 52(60%) male. Abdominal symptoms were present in 50%. CK7+/CK20- in 34(40%); CK7-/CK20+ in 2(2%) while CK7+/20+ in 7(8%) of metastatic tumor samples. Liver was metastatic site in 47(55%), Lymph node 12(14%) and Ascites in 8(9%). Endoscopy detected primary in 6 (7%) patients with 3 each in stomach and colon. No association of abdominal symptoms and cytokeratin markers was found with presence of GI primary site.

***Conclusion: ***Yield of localizing primary lesion in the GI tract by pan-endoscopy was limited. Abdominal symptoms and cytokeratin markers do not predict presence of gastrointestinal malignancies.

## INTRODUCTION

Carcinoma of unknown primary origin (CUP) is a heterogeneous group of cancers defined by the presence of metastatic disease with no identified primary tumor at presentation.^1^ It comprised 2% of all cancer in recent year and occurring mostly in 6th and 7th decade of life.^2^^,^^3^ The condition carries a poor prognosis with the average survival from 6 to 9 months.^4^ An extensive search to find the primary tumor is usually carried out. Even after extensive investigations that include imaging, endoscopies and immunohistochemistry studies the frequency of detection of a primary tumor is only upto 30%.^5^ Finding primary site not only have important bearing on therapeutic decisions by the physician^4^ but it also has prognostic implications.^6^ Some tumors especially metastatic colonic adenocarcinoma even at stage 3 and 4 has a 5 year survival ranging from 6% to 45%^7^ as opposed to hardly few months average survival for CUP.^5^

Guidelines and practices differ regarding the use of gastrointestinal (GI) endoscopies in finding the primary site. European Society for medical oncology (ESMO) guidelines^8^ recommend these endoscopies only if GI symptoms are present while National Comprehensive Cancer Network (NCCN) suggest esophagogastroduodenoscopy (EGD) / colonoscopy should be symptom directed and in addition to that colonoscopies should be performed in cases of adenocarcinoma or carcinoma unspecified metastasizing to the liver. EGD is also indicated if suspicion of finding primary in the upper GI tract is high based on the patient’s symptoms or other laboratory or radiological parameters.^8^^,^^9^ Immune-histochemistry with cytokeratin (CK) markers like CK 7 and 20 are routinely used for the tissue specimen. CK 7/20 positivity and/or negativity influences the decision to perform upper or lower GI endoscopies as they may direct towards the primary site in the GI tract.^4^^,^^8^^,^^10^

An invasive approach of EGD and colonoscopy must take into account the low yield of finding the primary. It therefore seems imperative to evaluate yield of these endoscopies in finding the primary site and whether any risk factors associated with presence of primary in GI tract. There is little data available related to yield of colonoscopy and EGD in determining the primary site in patients with CUP. The aim of our study was to evaluate the yield of colonoscopy and EGD in localizing primary tumor in patients with CUP. It also looked into the association of abdominal symptoms and CK 7 and 20 markers with the presence of GI primary.

## METHODOLOGY

***Patient and methods: ***This cross sectional study was conducted from December 2009 to December 2011 at The Aga Khan University Hospital on patients diagnosed as having biopsy proven CUP defined by the presence of metastatic disease with no identified primary tumor at presentation. The study protocol was approved by the Hospital Ethics review committee.

A total of 102 patients having CUP had colonoscopy and/ or EGD for GI symptoms, radiology and CK marker were enrolled after informed consent. EGD and colonoscopy were performed based upon the history, site, histopathology, tumor markers and radiology findings. Patients with unknown primary and having these procedures for upper GI bleed and perforation were excluded from the study. Similarly patients with deranged coagulation or on therapeutic anticoagulation were also excluded. Detailed history with particular emphasis on gastrointestinal related complaints like abdominal pain, bleeding per rectum and altered bowel habits were taken. Physical examination was carried out in every patient. All the data was collected on a predesigned questionnaire.

EGD and colonoscopies were done under conscious sedation with intravenous midazolam and fentanyl by gastroenterologists with Olympus GIF-Q 180 video scope and Olympus CF 180 AL Colonoscope respectively. Normal coagulation was assured before these procedures. Pulse, Blood Pressure and oxygen saturation were monitored before the start of procedure and every five minutes till completion. Details on preparation of colon, nature and location of the lesions were recorded. All lesions found at endoscopies were biopsied and sent for histo-pathological examination. Specimens were examined by at least two histopathologists before reporting.

***Statistical analysis:*** Descriptive statistics were calculated for continuous variables such as age, haemoglobin Mean ± SD were computed. For categorical variables such as gender, abdominal pain, biopsy site, histo-pathological type of lesion and radiological findings, the frequencies and percentages were calculated. The chi-square test was applied to assess the association of different variables like abdominal symptoms, cytokeratin markers with the primary lesion. P < 0.05 was considered significant. P value greater than this is reported as NS (Non-significant). Data entry and analysis were performed using the Statistical Packages for Social Sciences version 17(SPSS, Chicago, Illinois, USA).

## RESULTS

**Fig.1:**
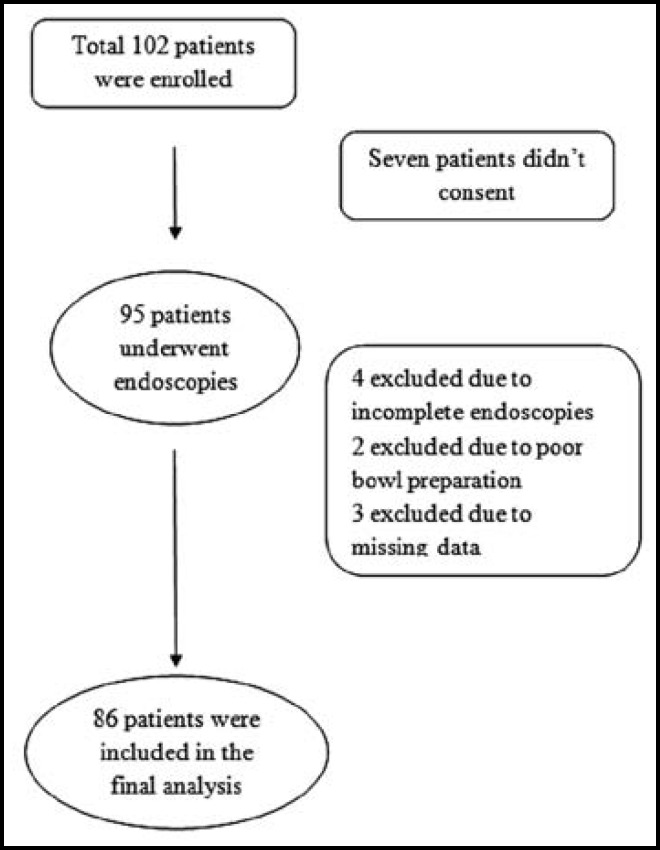
Flow Chart

A total of 102 patients of CUP were detected during study period. Out of which 86 patients underwent endoscopic procedures and were considered for final analysis. Detail of the excluded patients is given in Fig.1.

Of the 86 patients, the mean age of presentation was 55.10 +/- 11.94 years among which 71(82.5%) patients were from 41 to 69 years of age. The youngest patient was 23 years and the eldest was 85 years at the time of diagnosis. The male population comprised of 52 (60.46%) patients. Among all 86 patients abdominal pain, altered bowl habits and bleeding per rectum were present in 46.5%, 14% and 7%, respectively. The basic demographics along with the radiologic findings and CK markers distributions are given in Table-I.

The mean hemoglobin was 11.36 gms/dl having normochromic and normocytic picture. The predominant metastatic site was liver in 47(55%) patients followed by lymph nodes in 12(14%) patients, respectively. (Table-II) Among the 7(8.13%) bone biopsies five were from the vertebrae, while one each from scapula and right iliac crest. The soft tissue biopsy sites included buttock and a forearm nodule. Single metastatic deposit was in ovary, prostate and urinary bladder. The histopathology of the metastatic deposit was predominantly either adenocarcinoma or various sub-types of the same. (Table-III)

CK7 was positive in 34 (40%) while CK 20 was positive in 2 (2%) patients, respectively. CK 7/20 positive in 7 (8%) patients while both were negative in 43 (50%). Two patients with both CK7 and 20 positive were found to have malignancy in stomach and rectum, respectively. While among the two patients with CK7 positivity were found to have the primary in sigmoid and stomach respectively. The remaining two patients who were negative for both CK7 and CK20 had their primary found in stomach and transverse colon. The p-value for the cytokeratin markers with primary found in GI tract was found to be p = 0.46. (Table-IV)

All patients 86(100%) underwent colonoscopy while 60 (70%) patients underwent EGD in addition to colonoscopy according to the inclusion criteria. Primary tumor was detected in 6 (7%) patients. In three (3.48%) patients primary tumor was found in the stomach while in remaining three it was in colon. Among these three each has a malignancy in rectum, sigmoid and transverse colon respectively.

**Table-I:** Demographics, Radiological and Cytokeratin markers

*Demographics, Symptoms, radiological and tumor markers*	*n = 86 (%)*
*Gender*	
Male	52 (60.46 )
Female	34 (39.54 )
Age (years)	55.10 +/-11.94
Previous Malignancy	1 (1.1 )
Smoker	28 (32.55)
Alcohol	3 (3.48)
Weight loss	39(45.34)
Abdominal symptoms	43(50)
Hemoglobin (gms /dl)	11.36 ± 2.29
*Radiological findings*	
Ascites	28 ( 32.55)
Omental caking and mesenteric metastasis	26 (30.23)
Vertebral metastasis	17 (19.76)
Lymph nodes	37 (43.02)
Hepatic metastasis	56 (65.11)
*Cytokeratin markers*	
CK 7+/CK 20-	34(39.53)
CK 7-/CK 20+	2(2.32)
CK7+/CK20+	7(8.13)
CK 7-/CK 20-	43(50)

**Table-II:** Site of metastatic involvement

*Biopsy site*	*Frequency (%)*	*Primary on EDG*	*Primary on Colon*
Liver	47 (54.65%)	1	1
Lymph nodes	12 (13.89%)	0	0
Ascitic fluid	8 (9.30%)	0	0
Bone	7 (8.13%)	0	0
Omentum	5 (5.81%)	0	1
Soft tissue	2 (2.2%)	1	0
Pleural fluid	2 (2.2%)	1	0
Viscera	3 (3.48%)	0	1

**Table-III:** Histopathology of the metastatic site and their subsequent outcome on endoscopy

*Biopsy of metastatic site*	*Primary tumor not found*	*Primary found on EGD*	*Primary found on colon*	*Total (%)*
Adenocarcinoma	62	2	1	65 (75.58)
Poorly differentiated adenocarcinoma	4	1	1	6 (6.97)
Infiltrating adenocarcinoma	4	0	0	4 (4.65)
Neuroendocrine differentiation	3	0	0	3 (3.48)
Large cell carcinoma	2	0	0	2 (2.2)
Papillary carcinoma	2	0	0	2 (2.2)
Squamous cell carcinoma	1	0	0	1 (1.1)
Mucin secreting adenocarcinoma	1	0	0	1 (1.1)
Signet ring adenocarcinoma	0	0	1	1 (1.1)
Untype-able carcinoma	1	0	0	1 (1.1)
	80	3	3	86 (100%)

**Table-IV:** Correlation of abdominal symptoms and cytokeratin markers with the site of primary

	*Primary found at EGD*	*Primary found at Colonoscopy*	*P value*
Abdominal symptoms	1	2	0.717
CK 7-/20-	1	1	0.469
CK7+/20+	1	1	
CK7+/20-	1	1	

Out of the 86 colonoscopies 6(7%) patients had either an ulcer or a mass in their colon. Three (50%) out of these 6 patients with abnormal findings got their primary identified as a result of biopsy. Similarly out of 15 (17.4%) ulcers or a mass found at EGD, 3 (20%) turned out to be the source of primary.

Sub group analysis of the patients with adenocarcinoma of all categories along with unspecified carcinoma metastasizing to liver was done. There were a total of 44 patients among them 25 (56.8%) had abdominal symptoms while 18(41%) of patients were CK7-/20-. One primary was found out as a result of colonoscopy in this particular group. The p value for abdominal symptoms and cytokeratin markers with respect to primary found in colon was 0.451 and 0.806 respectively.

## DISCUSSION

This study showed a yield of around 7% in CUP by virtue of EGD and colonoscopy. However it failed to elaborate any co-relation between the different variables and EGD / colonoscopy with respect to this specific entity. The mean age of presentation in this study was 55 years as opposed to 59 years as reported from the literature from the West.^3^ In our study three patients were found to have cancer in the colon and in three patients the primary was found in the stomach. Overall there was no association between the abdominal symptoms and finding the primary in the gastrointestinal tract however two out of the three patients with primary found in the colon had abdominal pain.

In our study liver, lymph nodes and ascitic fluid were the most frequently involved sites for the metastatic disease respectively. In this regard Disibio et al in an autopsy based study concluded that lymph nodes are the most common site of metastasis (20.6%).^11^ Several studies have quoted different frequency of organ involvement depending upon the histo-pathological types of the disease.^12^^,^^13^

The expression of certain cytokeratin markers may help in differentiating the site of origin of different metastatic carcinomas. In our study, among three patients with metastatic colon cancers we found that CK 7 and 20 were both positive in one patient, both negative in another patient and the third patient had CK 7 positive with CK 20 negative. Previous studies have shown that CK 7 negativity with CK 20 positivity has the greatest predilection for colorectal cancers.^14^ However, it has also been demonstrated that upto 17% of colon cancers are positive for CK 7 whereas upto 19% of these tumors are negative for CK 20.^15^ Hence although CK7+/CK20- pattern in metastatic biopsy may point towards gastric cancer primary yet its absence does not rule out the possibility of primary colorectal neoplasm.^15^

In our study one out of three patients who had primary at stomach showed sole positivity for CK 7, none of the patients was sole CK 20 positive. However, single patient with CK7/20 positivity found to have primary residing in the stomach. This was also shown in study done by Pavlidis N.^10^ In contrast to colorectal adenocarcinoma; gastric adenocarcinomas have a heterogeneous expression of CK7/20. Studies have shown that CK 7-/20- pattern is seen in 10% gastric cancers while CK20+/7- pattern is seen in upto 33% of gastric adenocarcinomas while the rest exhibit mixed pattern.^14^^,^^16^^-^^18^ We also did not find any association of finding CK 7 or CK 20 with the detection of primary tumor. This is most likely because of small number of primary tumors in our cohort or because of heterogeneity of expression of these markers as observed by others as well.

It is interesting that there was no association of abdominal symptoms and detection of primary tumor in our study (p value NS). Studies have shown that patients with CUP mostly present with non specific symptoms of anorexia, weakness and weight loss. Early dissemination and lack of specific clinical features are hall mark of these malignancies. Moreover if there would have been prominent gastrointestinal symptoms those cases would have been detected earlier on with appropriate symptom directed investigations like colonoscopies and/or gastroscopies before their metastasis would come to lime light.

Sub group of patients having adenocarcinoma or carcinoma unspecified metastasizing to liver which underwent colonoscopy as indicated by NCCN guidelines, shows a poor yield of only one primary found as a result of colonoscopy in this sub select group of patient. Furthermore there was no correlation between the abdominal symptoms and the cytokeratin markers with respect to the primary found in the GI tract. However these findings can happen because of the poor yield of finding the primary in the GI tract.

We would like to highlight some of the limitations of this study. This was a single center tertiary care referral study. Our cohort consisted of small number of patients. But since CUP is not a very common disease it would be very difficult to recruit a large number of patients in a limited period of time. Endoscopies are operator dependent and carry a finite chance of missing a lesion depending upon bowl preparation and various other factors. Very limited number of patients had their primary found as a result of endoscopies. Hence getting a useful correlation in between the variables under consideration was difficult.

Further studies are required so as to potentiate findings depicted in this study particularly those with higher number of patients having their primary known. Specifically those in which not only symptom, immune-histochemistry and radiological findings are taken into consideration.

## CONCLUSION

The yield of finding a primary lesion by gastroscopy and colonoscopy is limited. Abdominal symptoms and cytokeratin markers do not seem to reliably predict the presence of colonic or gastric malignancies. More studies with a larger sample size are needed to further validate our findings.

## Authors contributions:

**TU** conceived and designed study, did literature search, collected and analyzed data. 

**AK** drafted initial manuscript and helped in recruitment of study patients. 

**HAS **was coordinator of the study. 

**TA **helped in drafting final manuscript. 

**HAS, SH and WJ** contributed in designing and supervising the study.
